# In rheumatoid arthritis inflamed joints share dominant patient-specific B-cell clones

**DOI:** 10.3389/fimmu.2022.915687

**Published:** 2022-07-27

**Authors:** Anne Musters, Giulia Balzaretti, Barbera D. C. van Schaik, Aldo Jongejan, Linda van der Weele, Sander W. Tas, Antoine H. C. van Kampen, Niek de Vries

**Affiliations:** ^1^ Department of Clinical Immunology & Rheumatology, Amsterdam University Medical Center (Location AMC)/University of Amsterdam, Amsterdam, Netherlands; ^2^ Laboratory of Experimental Immunology, Amsterdam University Medical Center (Location AMC)/University of Amsterdam, Amsterdam, Netherlands; ^3^ Bioinformatics Laboratory, Department of Epidemiology and Data Science, Amsterdam University Medical Center (Location AMC)/University of Amsterdam, Amsterdam, Netherlands

**Keywords:** rheumatoid arthritis, B-cell receptor, B-cell clones, synovial tissue, synovial tissue biopsy

## Abstract

**Background:**

In patients with rheumatoid arthritis (RA) different joints were shown to share the same dominant T-cell clones, suggesting shared characteristics of the inflammatory process and indicating that strategies to selectively target the antigen receptor might be feasible. Since T- and B-lymphocytes closely interact in adaptive responses, we analysed to what extent different joints also share dominant B-cell clones.

**Methods:**

In 11 RA patients, quantitative B-cell receptor (BCR) repertoire analysis was performed in simultaneously obtained samples from inflamed synovial tissue (ST) from distinct locations within one joint, from multiple joints, from synovial fluid (SF) and peripheral blood (PB).

**Results:**

ST biopsies from different locations in the same joint showed clear overlap in the top-25 dominant BCR clones (16.7%, SD 12.5), in the same range as the overlap between ST and SF in the same joint (8.0%, SD 8.8) and the overlap between ST-ST between different joints (9.1%, SD 8.2), but clearly higher than the overlap between ST and PB (1.7%, SD 2.4; p<0.05) and SF and PB (2.7%, SD 4.1; p<0.05). Interestingly, these figures were substantially lower than the overlap observed in previous T-cell clonality studies.

**Conclusions:**

We conclude that in RA BCR clonal responses may be more localized than TCR clonal responses, pointing to antigen-selective influx, proliferation and/or maturation of B-cells. B lineage cells in the SF may adequately represent the dominant BCR clones of the ST, which is in contrast to T-cells. Collectively, the presence of shared B- and especially T-cells in different joints from the same patient suggests that approaches might be feasible that aim to develop antigen-receptor specific targeting of lymphocyte clones in RA as an alternative to more generalized immunosuppressive strategies.

## Introduction

Adaptive immune cells are key players in the pathogenesis of rheumatoid arthritis (RA) (1–8). The immune response in RA is encoded by rearranged receptors expressed by clones of T and B lymphocytes, plasmablasts and plasma cells. Targeting the adaptive immune response using novel targeted therapies, also known as biologics, for instance with abatacept (CTLA4-Ig) or rituximab has been proven to be of clinical benefit in RA patients (6, 9). Despite these important developments in the treatment of RA, there is still no cure for RA patients available. Only 60% of RA treatments are effective, and often merely induce a partial clinical response (10). Thus, there is a clear need to develop novel, targeted, more effective therapies.

Recent studies showed that a common T-cell receptor signature can be found in the synovial tissue (ST) of RA patients (11). This suggests that antigen- and/or receptor-specific therapies targeting a limited set of T-cell clones in individual patients may be feasible in RA. Another key player of the adaptive immune system, the B-cell, also appears to be of great relevance in RA as it is an antibody-driven disease in which anti-cyclic citrullinated peptide antibodies (ACPA) and IgM-rheumatoid factor (IgM-RF) play a key role. Consequently, antigen receptor specific targeting of B-cells might even be more attractive. However, to assess whether B-cells might indeed constitute an interesting target for antigen receptor specific therapy, more information on the distribution of B-cell clones over different joints in the same patient is required.

This study was designed to investigate the distribution of the B-cell receptor (BCR) repertoire using a high-throughput quantitative approach in a unique cohort of 11 RA patients, in which synovial tissue (ST) biopsies were taken from multiple locations within the same joint, in another (contralateral) joint, as well as synovial fluid (SF) and peripheral blood (PB) samples. Using this method, we aimed to answer the following three questions: 1) Do different B-cell clones dominate the BCR repertoire at different locations within one single inflamed joint? 2) Are BCR repertoires in different inflamed joints dominated by the same BCR-clones? 3) Do the same dominant BCR-clones reside in different compartments, such as ST, SF and PB at the same time?

## Materials and methods

### Patients

Eleven RA patients meeting the 2010 ACR/EULAR Classification Criteria for RA with active disease (disease activity score evaluated in 28 joints (DAS28) >3.2) were included (12). Details of the included patients are described earlier (11). All were autoantibody positive (anti-cyclic citrullinated peptide test >25 kAU/l and/or IgM-rheumatoid factor >12.5 kU/l). Two patients (patients 3 and 11) were treated with a biological at the time of arthroscopy, i.e. rituximab and infliximab (both last infusion 1 month before sampling). More details on patient characteristics are shown in **Table 1**. From 7 patients synovial tissue biopsies were taken from two inflamed joints, all paired with peripheral blood. From 4 of these patients we also collected synovial fluid from the biopsied joint prior to the arthroscopy. Three additional patients were included for paired SF and PB analysis. The study was approved by the independent Medical Ethics Committee of the Amsterdam University Medical Center/University of Amsterdam and performed according to the Declaration of Helsinki. All patients gave written informed consent.

**Table 1:** Patient characteristics (n=11).

Age (mean (SD)), years	53 (13)
Male (n (%))	2 (18%)
IgM-RF positive (n (%))	11 (100%)
IgM RF level (median (IQR)), kU/L	68 (33-322)
ACPA positive (n (%))	6 (55%)
ACPA level (median (IQR)), kAU/L	84 (8-499)
IgM-RF and ACPA both pos. (n (%))	6 (55%)
Disease duration (median (IQR)), months	45 (3-192)
DAS28 (mean (SD))	5.2 (1.2)
Patients without therapy (n (%))	7 (64%)
Patients treated with csDMARD (n (%))	4 (36%)
Patients treated with bDMARD (n(%))	2 (18%)

IgM-RF, IgM rheumatoid factor; ACPA, anti-citrullinated peptide antibodies; csDMARD, conventional synthetic disease-modifying antirheumatic drugs; bDMARD, biological disease-modifying antirheumatic drugs.

### Sampling of synovial biopsies, synovial fluid and peripheral blood

To obtain ST biopsies a minimally invasive arthroscopy was performed from a clinically inflamed knee or ankle, as described previously (13). From 12 out of the 15 inflamed knee joints biopsied, ST biopsies were taken from two locations, the infrapatellar (IP) and the suprapatellar (SP) region. In all patients who underwent arthroscopy, both knee joints where biopsied for ST on the same day. SF was obtained by arthrocentesis. In case of a combined collection of ST and SF, the SF was collected prior to the arthroscopy in order to avoid contamination of SF by hemorrhagic fluid. In all patients peripheral blood was drawn at the time of the arthroscopy and/or arthrocentesis.

### Linear amplification and next-generation sequencing

The protocol used for linear amplification and next-generation sequencing of the BCR-heavy chain (BCR-heavy) was based on previously described methods (8, 11, 14–16). Sequencing was performed on the MiSeq (Illumina). To identify and quantify separate BCR-clones more accurately unique molecular identifiers (UMI) were used (17). A unique clone was identified by the unique sequence of the CDR3-region in combination with the use of the (V)ariable and (J)oining genes. Based on earlier studies BCR-clones with a frequency ≥ 0.5% were considered dominant, and therefore called Highly Expanded Clones (HECs) (8, 18). BCR repertoire raw fast data are available on the Sequence Read Archive with the accession number BioProject PRJNA822925.

### Bioinformatics and statistics

Values are either expressed as mean or median depending on the presence of a (non)-normal distribution of the data. For each sample an equal number of reads (N=9,736) was randomly drawn from the acquired reads to standardize comparisons. The Chao-modified Sørensen index was used to test for similarity between different samples. This biodiversity index measures dispersion and gives a value between 0 and 1. Values near 0 indicate no overlap between two locations, while values close to 1 show that the two repertoires are identical (19–22). Because somatic hypermutation causes a broader, and more diverse repertoire we decided to only use the CDR3-sequence for the similarity analysis of the top clones and total repertoire. Differences between groups were analyzed using the unpaired Mann–Whitney U test, paired t test, one-way ANOVA or Tukey’s multiple comparison test where appropriate. p Values < 0.05 (two-sided) were considered statistically significant. R (version 3.5.1), package SpadeR (version 0.1.1) and Graphpad Prism (version 8.0) were used to perform the analyses.

## Results

### Different locations within an inflamed joint share dominant BCR clones

When performing an arthroscopy the knee joint can be divided into two anatomic locations, the suprapatellar (SP) and infrapatellar (IP) region. For T-cells we know that synovial tissue biopsies from different locations within the same joint show substantial clonal overlap (11). However, for B-cells this is still unknown. In this study we took biopsies from these distinct two regions from 12 different inflamed knee joints in 7 individual RA patients.

**Figure 1:**
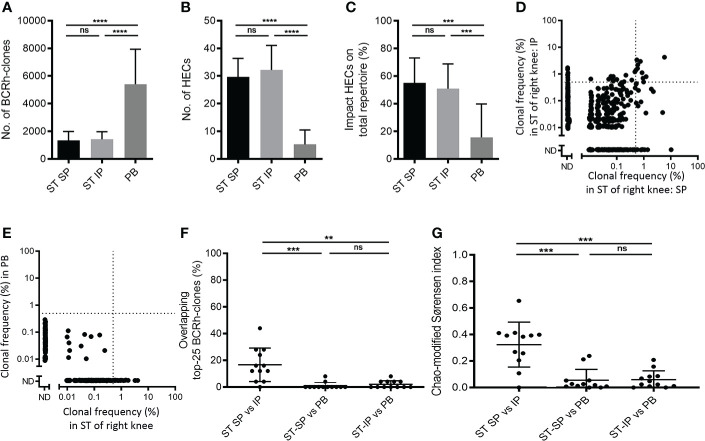
Comparing B-cell receptor repertoires within one joint. Bar charts of **(A)** the number of BCR-clones, **(B)** number of highly expanded BCR-clones (HECs) and **(C)** impact of HECs on total repertoire per compartment (bars show mean and SD; ** p<0.01 and **** p<0.0001 using a Tukey’s multiple comparison test; ns=not significant). **(D)** Representative example of overlap-plot in one patient comparing the suprapatellar (SP) to the infrapatellar (IP) synovial tissue (ST) region, showing overlap of dominant BCR-clones in the upper right quadrant. Each dot reflects the frequency of the clone (% of total number of reads) in the ST-IP on the Y-axis and in the ST-SP on the X-axis. Vertical and horizontal dotted lines indicate the 0.5% threshold for HECs. ND=not detected; **(E)** Representative example of a comparison of ST to peripheral blood (PB), showing little overlap. For explanation of graph see **(D)** above. Scatter plots of **(F)** percentage of overlapping top-25 BCR-clones and **(G)** Chao-modified Sørensen indices of the total BCR-clones repertoire when comparing different compartments (n=12; lines at mean and SD; * p<0.05, ** p<0.01 using a paired t test; ns=not significant).

The ST biopsies were analyzed for BCR repertoires and compared with paired peripheral blood (PB) samples. The SP and IP regions showed comparable numbers of BCR-clones (mean ± SD: 1,337 ± 651 versus 1,420 ± 552 respectively) and highly expanded BCR-clones (HECs; mean ± SD: 29.7 ± 6.7 versus 32.2 ± 8.9), with a comparable impact of these HECs on the total repertoire (mean ± SD: 55.1% ± 18.0 versus 50.8% ± 17.9). In contrast, PB showed a significantly higher number of BCR-clones (p < 0.0001; 5,407 ± 2,533), a lower number of HECs (p < 0.0001; 5.3 ± 5.2), and these HECs accounted for significantly less impact on the total repertoire (p < 0.0001; 15.6% ± 24.1) (**Figures 1A**–**C**).

In order to determine similarity between different BCR repertoires we compared the most expanded BCR-clones between the two biopsied regions, using PB as control. Of the 25 most expanded BCR-clones in the SP region on average 16.7% (mean, SD 12.5) were also present in the top-25 clones in the IP region (“Clonal retrieval”). However, significantly fewer of the expanded BCR clones in SP (p<0.001; 1% ± 2.5%) and IP (p<0.01; 2% ± 2.7%) could be retrieved in PB samples (**Figures 1D**–**F**).

To analyze similarity for the complete BCR, rather than the most dominant clones only, we use the Chao-modified Sørensen index (11). This index gives an estimate for the combined frequency of all shared clones while correcting for shared clones not observed due to incomplete sampling. The comparison of the SP and IP region showed a mean similarity index of 0.32 (SD 0.17; n=12), significantly higher than the score of 0.06 observed when comparing ST-SP or ST-IP with PB (mean, SD 0.08 and 0.07 respectively, n=12; p<0.001, **Figure 1G**).

Thus, the BCR repertoire in inflamed ST of RA patients shows similarity at different locations within same joint, and shows sharing of some dominant BCR clones, whereas hardly any overlap was observed when comparing ST to PB.

### BCR repertoires in different inflamed joints share distinct dominant BCR-clones

**Figure 2:**
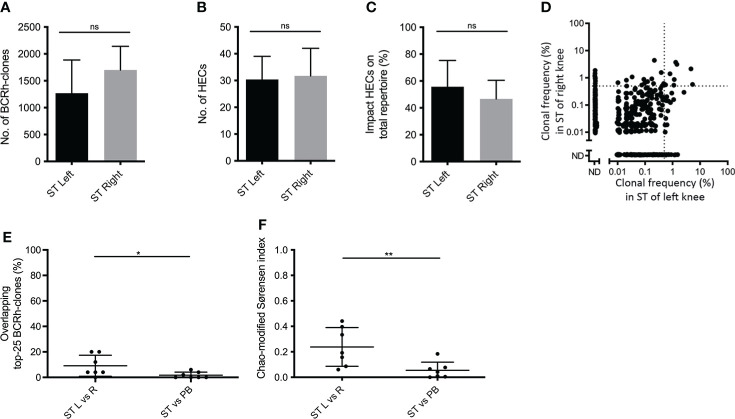
Comparing B-cell receptor repertoires in two contralateral inflamed joints. Bar charts of **(A)** the number of BCR-clones, **(B)** number of highly expanded BCR-clones (HECs) and **(C)** impact of HECs on total repertoire per joint (bars show mean and SD; ns=not significant using a one-tailed Mann-Whitney test). **(D)** Representative example of overlap-plots from one patient when comparing the ST of the left (L) joint to right (R) joint, showing substantial overlap; for explanation see legend **Figure 1D** **(E)** Scatterplot of the impact of the top-25 overlapping BCR-clones and **(F)** the Chao-modified Sørensen indices of the total BCR-clonal repertoire when comparing different compartments (n=7; lines at mean and SD; * p<0.05, ** p<0.01 using a paired t test).

To test for sharing of clones between different joints in 7 RA patients the BCR repertoire was compared between ST biopsies from inflamed contralateral knee joints in the same patient that were taken on the same day. Again, paired PB samples from the same time-point served as a control. The overall repertoire characteristics from contralateral joints were not significantly different (**Figures 2A**–**C**): In the left knee on average 1,269 ± 616.5 BCR-clones, with 30.4 ± 8.6 HECs accounting for 55.8% ± 19.5% of the total repertoire, in the right knee 1,701 ± 439.7 BCR-clones, with 31.7 ± 10.3 HECs accounting for 46.7% ± 13.9 of the total repertoire.

To test for repertoire similarity between different joints we compared the overlap between the 25 most expanded BCR-clones in both knee joints. The overlap of the top-25 between two different joints was 9.1% ± 8.2, significantly larger than the top-25 overlap between ST and PB (1.7% ± 2.4; p = 0.02; **Figures 2D**, **E**). Comparing the total repertoires, the mean Chao-modified Sørensen index was 0.24 ± 0.15 for the ST-ST comparison, significantly higher than the index between ST and PB (0.05 ± 0.06; p < 0.01; **Figure 2F**). Both the top-25 overlap and Chao-modified Sørensen index between contralateral joints did not differ from the overlap observed when comparing two ST-regions within one joint (p = 0.08 and p = 0.36 respectively).

Together, these data demonstrate that the BCR repertoire in ST from the knee shows substantially more overlap with the repertoire in ST of the contralateral knee than with PB.

### BCR-clones that are dominant in ST are shared with SF and to a lesser extent with PB

The pathogenesis of arthritis is often studied by looking into the SF rather than ST. However, until recently, it was still unclear whether the adaptive immune responses in both compartments was similar. We have learned from an earlier study on T-cells that the dominant TCRβ-clones in ST are not fully reflected in SF and even less so in PB (11). However, for B-cells this was not investigated before. Therefore, we included 6 RA patients from whom we obtained simultaneously obtained samples: ST biopsies and SF from the same joint, paired with PB as control.

**Figure 3:**
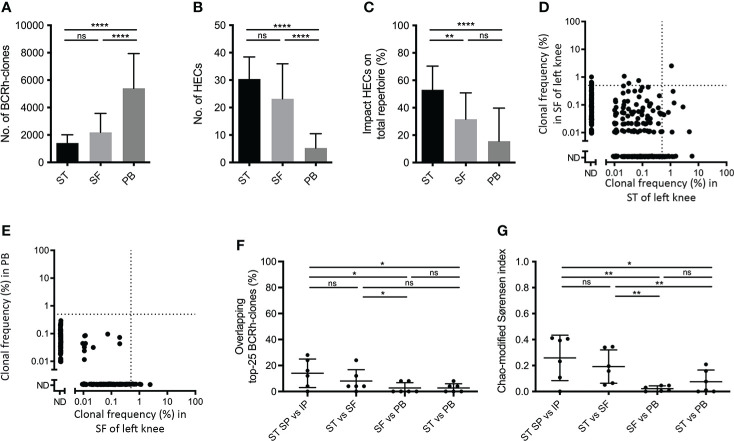
Comparing B-cell receptor repertoires in synovial tissue, synovial fluid and peripheral blood. Bar charts of **(A)** the number of BCR-clones, **(B)** number of highly expanded BCR-clones (HECs) and **(C)** impact of HECs on total repertoire per compartment (bars show mean and SD; ** p<0.01, **** p<0.0001, using a Tukey’s multiple comparison test; ns=not significant). **(D)** Representative overlap-plots in one patient comparing the synovial tissue (ST) to synovial fluid (SF) and **(E)** comparing the SF to peripheral blood (PB) in one patient; for further details see legend **Figure 1D**. Scatter plot of **(F)** percentage of overlapping top-25 BCR-clones and **(G)** Chao-modified Sørensen indices of the total BCR-clones repertoire when comparing different compartments (n=6; lines at mean and SD; ** p<0.01, *** p<0.001 using a paired t test; ns=not significant).

In ST and SF we detected a comparable number of BCR-clones (1,412 ± 598 vs. 2,188 ± 1,385) and HECs (30.4 ± 8.0 vs. 23.2 ± 12.8; **Figures 3A**, **B**). However, the impact of the HECs on the repertoire was significantly higher for ST compared to SF (p < 0.01; 53.1% ± 17.3 versus 31.7% ± 19.2; **Figure 3C**). ST and SF differed significantly from PB regarding the number of BCR-clones and HECs (both p <0.0001), while the impact of HECs on the total repertoire was only significantly different between ST and PB (p < 0.0001; **Figures 3A**–**C**).

The top-25 overlap in BCR clones between SF and ST from the same joint was 8.0% ± 8.8, significantly higher than the 2.7% ± 4.1 top-25 overlap between SF and PB (p < 0.05; **Figures 3D**–**F**). This top-25 overlap did not show significant differences in the comparisons ST-SF vs. ST-PB, or SF-PB vs. ST-PB. However, comparing all BCR-clones the Chao-modified Sørensen index was 0.20 ± 0.13 for the SF-ST overlap, significantly higher than the index of 0.02 ± 0.02 for the SF-PB comparison and 0.05 ± 0.06 for the ST-PB comparison (both p < 0.01; **Figure 3G**). Of note, the intrajoint overlap scores did not significantly differ between ST-ST and ST-SF, not when using the top-25 overlap and neither when using the Chao-modified Sørensen index (**Figures 3F**, **G**).

## Discussion

Using quantitative, B-cell receptor repertoire analysis we demonstrate that RA synovitis in different joints shares dominant B-cell responses. Within the same patient a limited number of expanded B-cell receptor clones were retrieved in the inflamed synovial tissue and fluid in different joints. The observed sharing between synovial tissue in different joints suggests that immunotherapy, selectively targeting a limited number of shared, expanded B-cell clones might be feasible and effective (23)

However, compared to the top-25 overlap observed in T-cell clones within and between synovial tissue samples taken in the same (54%) and in different joints (50%) in a previous study), the overlap in the B-cell compartment is much less pronounced (17% and 9%, respectively) (11). This is even more clear in the comparison of synovial tissue with peripheral blood (20% for T-cell and 2% for B-cell clones). This suggests that B-cell responses might be much more localized than T-cell responses. This might suggest localized proliferation and/or maturation with an increase in BCR expression, e.g. in the transformation of mature B-cells to plasma cells. Another explanation could be an antigen-driven influx of specific B-cell clones. Such a localized influx of B-cells into the joint on a stable TCR background might result in a clinical arthritis. It would be very interesting to further investigate this into more detail, e.g. in a prospective study in seropositive individuals at increased risk of RA while sampling paired synovial tissue, fluid and blood samples during the course of the disease.

It is remarkable that in the BCR repertoire the observed ST-SF overlap (8%) is actually in the range of ST-ST overlap within and between different joints (resp 17% and 9%), while the earlier study for T-cell overlap demonstrated a significantly higher ST-ST overlap than SF-ST overlap (p < 0.01; 54% and 50% vs 26%, respectively). This might indicate that there is more circulation of B-cells than T-cells from the synovium to the synovial fluid in the B-cell compartment than in the T-cell compartment. Since intra-joint overlaps observed in the ST-SF and ST-ST comparisons are in the same (albeit low) range, our data suggest that B cells derived from synovial fluid or alternatively from synovial tissue are equally informative regarding antigen specificity of B-cells in the synovium. This indicates that for future B-cell studies in RA one might use SF from arthrocentesis as a substitute for ST obtained by arthroscopy or ultrasound-guided biopsy. The latter may have several advantages since arthrocentesis is a less invasive, patient-friendly procedure, which does not require theatre time.

More studies are required to further characterize these overlapping TCR and BCR clones. This might entail functional characterization, including antigen specificity, in order to develop antigen receptor directed therapies. However, it might also concern genomic and proteomic analyses in order to identify more uniform cellular targets selectively present in the activated B- and/or T-cell clones. Although isolation of B-cells from synovium without modification of the expression of surface markers is challenging, it is important to commence these analyses in the near future in order to develop more selective immunotherapy.

We wish to note that in our efforts to perform a UMI-standardized reproducible quantitative analysis of the BCR repertoire we based our analysis on mRNA of the heavy chain only. One might argue that different cell types from the B-cell lineage, such as B-cells and plasma cells, express different levels of BCR-heavy mRNA. Literature describes a 5-50 fold difference in the expression of BCR mRNA in plasma cells and B-cells, obviously dependent on the activation status of the B-cell (24, 25). As noted above, the observed BCR clonality might thus in part reflect selective maturation rather than proliferation in the individual joint. We do not think that the fact that our analysis is only based on heavy chain analysis renders our results less valid: the enormous variability in the heavy chain produced by somatic rearrangement and mutation is sufficient to distinguish the different clones for the purpose of our clonality analysis. However, for further functional assays identification of the linked light chain is clearly desirable, e.g. using single cell analysis.

In conclusion, BCR repertoires in biopsies from inflamed ST from different sites within one joint, from different joints and from SF share dominant BCR clones, while these clones have very low frequencies in PB. This suggests that for some B-cell (receptor) analyses both tissue biopsies and synovial fluid may be equally informative, the latter being more patient-friendly. Our results show that shared underlying B-cell responses might underly inflammation in different joints. This supports the idea that antigen- and/or receptor-specific therapies targeting a limited set of B-cell and/or T-cell receptor clones in individual patients might be feasible in rheumatoid arthritis or even in the “immune-mediated inflammatory diseases”-group at large.

## Data availability statement

The datasets presented in this study can be found in online repositories. The names of the repository/repositories and accession number(s) can be found below: NCBI, accession ID: PRJNA822925.

## Ethics statement

The studies involving human participants were reviewed and approved by Medical Ethics Committee of the Amsterdam University Medical Center/University of Amsterdam. The patients/participants provided their written informed consent to participate in this study.

## Author contributions

AM, ST, and NV contributed to conception and design of the study. AM organized the database. GB performed the linear amplification and next-generation sequencing. BS, AJ, and AK performed the biostatistics. AM, GB, and NV performed the statistical analysis. AM wrote the first draft of the manuscript. AM and NV wrote sections of the manuscript. All authors contributed to manuscript revision, read, and approved the submitted version

## Funding

This work was supported by the IMI-funded project BeTheCure (grant No 115142-2).

## Conflict of interest

The authors declare that the research was conducted in the absence of any commercial or financial relationships that could be construed as a potential conflict of interest.

## Publisher’s note

All claims expressed in this article are solely those of the authors and do not necessarily represent those of their affiliated organizations, or those of the publisher, the editors and the reviewers. Any product that may be evaluated in this article, or claim that may be made by its manufacturer, is not guaranteed or endorsed by the publisher.
